# *ACME: Automated Cell Morphology Extractor* for Comprehensive Reconstruction of Cell Membranes

**DOI:** 10.1371/journal.pcbi.1002780

**Published:** 2012-12-06

**Authors:** Kishore R. Mosaliganti, Ramil R. Noche, Fengzhu Xiong, Ian A. Swinburne, Sean G. Megason

**Affiliations:** Department of Systems Biology, Harvard Medical School, Boston, Massachusetts, United States of America; Carnegie Mellon University, United States of America

## Abstract

The quantification of cell shape, cell migration, and cell rearrangements is important for addressing classical questions in developmental biology such as patterning and tissue morphogenesis. Time-lapse microscopic imaging of transgenic embryos expressing fluorescent reporters is the method of choice for tracking morphogenetic changes and establishing cell lineages and fate maps *in vivo*. However, the manual steps involved in curating thousands of putative cell segmentations have been a major bottleneck in the application of these technologies especially for cell membranes. Segmentation of cell membranes while more difficult than nuclear segmentation is necessary for quantifying the relations between changes in cell morphology and morphogenesis. We present a novel and fully automated method to first reconstruct membrane signals and then segment out cells from 3D membrane images even in dense tissues. The approach has three stages: 1) detection of local membrane planes, 2) voting to fill structural gaps, and 3) region segmentation. We demonstrate the superior performance of the algorithms quantitatively on time-lapse confocal and two-photon images of zebrafish neuroectoderm and paraxial mesoderm by comparing its results with those derived from human inspection. We also compared with synthetic microscopic images generated by simulating the process of imaging with fluorescent reporters under varying conditions of noise. Both the over-segmentation and under-segmentation percentages of our method are around 5%. The volume overlap of individual cells, compared to expert manual segmentation, is consistently over 84%. By using our software (ACME) to study somite formation, we were able to segment touching cells with high accuracy and reliably quantify changes in morphogenetic parameters such as cell shape and size, and the arrangement of epithelial and mesenchymal cells. Our software has been developed and tested on Windows, Mac, and Linux platforms and is available publicly under an open source BSD license (https://github.com/krm15/ACME).

This is a *PLoS Computational Biology* Software Article.

## Introduction

Pattern formation and tissue morphogenesis are two classical and unsolved problems in developmental biology. Patterning refers to the process by which the embryo generates the right kind of cells at the right place and time. Morphogenesis refers to how tissues are bent and molded to achieve the right shape and form. Modern systems-based approaches to understand these processes *in vivo* involve using advanced imaging techniques to elucidate how mechanisms at multiple spatial scales i.e., molecular networks, single cell behaviors, cell-cell interactions, and tissue mechanics, are coordinated to turn an egg into an embryo [1], [2]. By systematically imaging embryos expressing fluorescent proteins with confocal or two-photon microscopy (*in toto* imaging), one can watch events at cellular resolution and then quantitatively model these events inside a computer [3].

In toto imaging generates large quantities of images depicting developmental dynamics in the embryo across space and time [4]–[6]. For example, a confocal or two-photon imaging session can capture three-dimensional images covering a field-of-view of 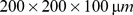 with a spatial sampling of 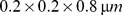 and with a time-sampling rate of 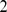 minutes over a period of 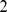 days. The process of imaging consists of irradiating the specimen with laser light focused on successive optical planes in 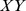. The useful sampling interval between successive optical planes is limited by the point-spread function (PSF) of the optics leading to worse resolution and thus larger sampling intervals along the 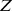-axis in comparison to the 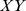 plane. Such an imaging experiment typically generates 100,000 images per experiment, with about 5000 cells in a given 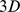 image and over 100,000 cell tracks and division events in the whole dataset. As a result, automated image analysis techniques are essential for extracting cell kinematic and morphogenetic parameters such as cell shapes, cell trajectories, cell packing, and tissue rearrangement patterns [6]–[8]. Automatic extraction needs to be robust since manual curation of errors is laborious even at low error rates for a large field of cells.

Over the past decade, a number of automated methods were developed for 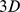 nuclei-specific segmentation including watershed [9]–[11], active surface based methods [12]–[16] and gradient vector flow methods [17]. However, robust segmentation of membranes rather than just nuclei remains a difficult problem. Most techniques for membrane segmentation use nuclear segmentations as seeds for expanding into membranes [12], [16]. The reason progress on segmentation algorithms for membrane has lagged behind nuclei is manifold: cell nuclei are better separated; have more consistent and simple shapes; maintain a condensed marker expression, and are more photostable for time-lapse experiments. However, in many situations, nuclear images require additional acquisition overhead and membrane information may be more vital in a study. For example, membranes are pertinent to the analysis of cell behavior and morphogenesis since cell shape and size, and intercellular contact areas can be directly quantified. Thus, there is a compelling need for algorithms that obtain membrane segmentations directly when there are no nuclear images available.

To address this need, we present a fully automated method with corresponding open-source, cross-platform software (ACME) to reconstruct weak membrane signals for achieving high-quality cell segmentations. We validated our algorithm using synthetically generated images for which ground-truth is known as well as with real images that were manually segmented by an expert. For generating synthetic data, we developed novel simulations of the image acquisition process replete with suitable noise models. Using simulated data, the performance of the algorithm was comprehensively evaluated against different noise conditions. To further demonstrate the utility of our method, we quantified cell shape and size, and the development of epithelial and mesenchymal characteristics in images of the zebrafish presomitic mesoderm. Our algorithm enabled us to quantify differences in the dynamics of cell sub-populations that correlate with the mesenchymal to epithelial differentiation process. Our methods are computationally-efficient, powerful, and widely-applicable to the quantitative analysis of cell dynamics during morphogenesis.

## Design and Implementation

### Membrane signal reconstruction for accurate image segmentation

**Figure 1:**
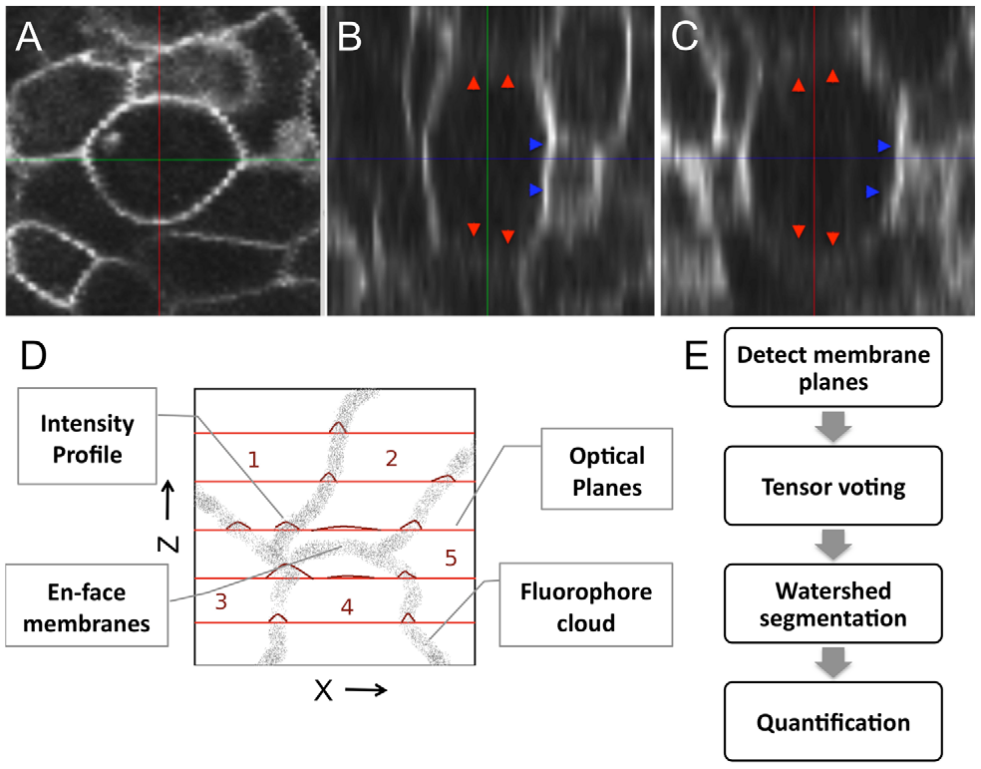
Reconstructing the membrane signal by eliminating intensity inhomogeneities. A single cell membrane is shown across (A) 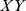, (B) 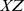, and (C) 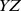 sections. The 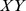 plane shows a consistently bold and uniform membrane signal while the 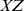 and 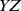 views show a non-uniform membrane signal. Membrane planes *en-face* to the 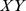 optical planes (marked by red arrows) are very weak and markedly diffuse in intensity. Membranes orthogonal to the 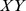 imaging plane are sharper (blue arrows). (D) A qualitative model describing the formation of a membrane image under a fluorescent microscope. Flourophores tagged to membranes are shown as a point cloud (input). The 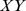 focal planes are shown in red and the obtained intensity profiles on the plane are shown as plots. Cell membranes imaged oblique and *en face* such as the interface between cells 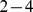 are poorly visible in comparison to those orthogonal to the focal planes. (E) Three stages in the reconstruction process: (i) Detect membrane planes by mining for planar fluorophore distributions. This allows even weak membranes (*en-face* or oblique) to be extracted and accounts for intensity inhomogeneity. (ii) Voting to fill structural gaps or holes in the membrane signal that may not be contiguous. (iii) Region segmentation using the watershed algorithm to extract three-dimensional cell meshes for quantification.

Two big challenges with membrane data are the presence of intensity inhomogeneities and punctuated gaps along the three-dimensional boundary. In Figure 1(A–C), we show a single cell membrane across the three cross-sections of 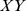, 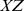 and 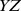. Intensity inhomogeneity (red and blue arrows) can be explained with help of an image formation model for membranes (Figure 1(D)). Here, optical planes (red lines) periodically section a dense cloud of fluorescent proteins tagged to membranes. The point spread function (PSF) of the optics accumulates emissions from a small neighborhood of fluorophores and creates intensity profiles shown in dark red. Mathematically, this is a convolution of the PSF with the fluorophore density function of the sample. The intensity at a voxel is therefore representative of the fluorophore density at the focused region of the tissue. Cell junctions are generally more intense as a result of high spatial concentration of fluorophores arising from the co-localization of multiple membranes. Additionally, membranes that are orthogonal to the imaging planes depict a crisp and bright intensity profile, whereas oblique membranes appear diffuse (observable in the 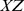 and 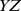 views in Figure 1(B–C)). This is because the PSF, as mentioned before, is shaped similar to an anisotropic Gaussian kernel with 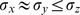. The strength of the output signal is thus dependent on the relative alignment of he membrane and the PSF kernel. Thus, orthogonal membranes present a strong signal because the PSF samples fluorophores in the membrane “above” and “below” the focal plane. For oblique membranes, the space “above” and “below” is non-fluorescing so that output signal is weaker. In the limit, *en-face* membranes, especially those in between imaging planes are often very dim and difficult to detect even by the human eye.

In order to correct these two problems, we developed signal reconstruction techniques. Our algorithms are inspired by work on vessel-detection from MR and CT imagery in which Hessian-based filters were designed to detect vessels [18], [19]. They used the fact that eigenvectors of the image Hessian point in the directions of principle curvature. At vessel boundaries, the eigenvector with largest eigenvalue is almost normal to the boundary, and the one corresponding to the smallest eigenvalue points along the vessel axis. Here we extend these ideas to planar membrane structures and further improve these results using a voting strategy.

Our method has three stages (Figure 1(E)): (i) We observe that membranes assume locally linear intensity patterns especially in dense cell regions. This is used to design image processing filters to identify planar intensity formations in images. Automated scale-selection is accomplished by identifying the relative orientation of the membrane planes with a putative Gaussian PSF. (ii) Near inter-cellular junctions and due to image noise, the planarity assumption breaks down causing structural gaps to appear. The identified planar components are then used in a voting framework to fill gaps and eliminate spurious structures. (iii) After reconstructing membrane planes, we use the popular watershed methodology for image segmentation to identify cells.

### Planarity detection function for locating membrane planes

Using our preliminary work in [20], we designed an image filter that responds to 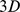 locally planar intensity structures such as those found in cell membranes and suppresses all other types of intensity patterns. The derived filter is based on the the Hessian matrix (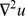) of the intensity function 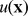 combined with normalized Gaussian derivatives that provides an aspect of scale. Let 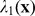, 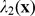 and 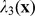 be the sorted (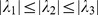) eigenvalues of 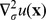 with corresponding eigenvectors 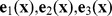.

**Figure 2:**
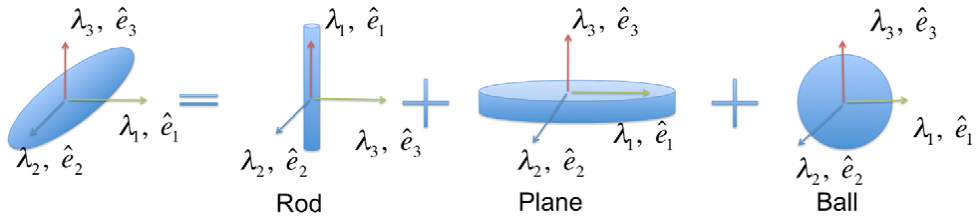
Tensor decomposition. A symbolic illustration of a generic tensor represented in terms of basis tensors of type plane, rod and ball.

In a local coordinate reference frame placed at a membrane voxel, we are interested in identifying the neighborhood intensity distribution. There are three types of distribution shapes that can be detected: rod, plane and ball (Figure 2). Based on our presentation, it is easy to see that a rod has a 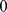 change in second derivative of intensity along its axis and maximal change in the cross-sectional plane. Hence, 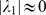 and 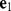 points along the axis. 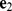 and 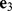 lie in the cross-sectional plane with 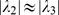. In the case of a plane, the maximal change in directional second derivative is along the normal and there is no change in the plane. Hence, we have 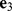 along the normal and 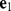 and 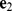 lying on the plane. Correspondingly, the eigenvalues follow the relation 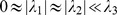. Finally, for a ball (uniform signal), there is no preferential orientation and all directions have the same change of directional second derivatives. Hence, 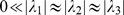. Table 1 summarizes these different cases.

**Table 1:** Geometric structure classification based on eigen-system.

Structure	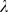	A	B	S
Foreground	-	-	-	high
Plane	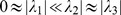	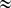 1	0	high
rod	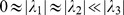	0	0	high
Ball	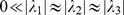	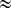 1	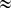 1	high
Background	-	-	-	low

An overview of the local intensity structures determined by their eigen-system. Parameters **A**, **B**, and **S** refer to individual terms in the planarity filter (Equation 1 and 2). These terms are specified as ratios of individual eigenvalues to enhance the identification of planes relative to rods and ball structure classes.

In order to detect membrane structures, we want to selectively identify pixels that belong to a plane distribution rather than a ball or a rod. Hence, we define the *planarity* of a voxel 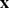 as the similarity of the local neighborhood 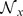 to a plane-like structure, as:
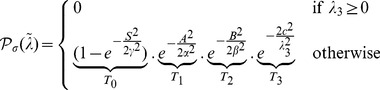
(1)

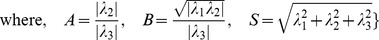
(2)


Here, 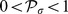 with larger values indicating more similarity to a plane at regularization scale 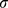. The free parameters 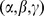 are set to 1 in our experiments and code but may be fine-tuned depending on the specifics of the imaging modality. For the case of bright membranes on a dark background, a positive 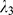 denotes background and hence the planarity output is set directly to 0. We now explain how background voxels and voxels corresponding to the rod and ball forms are suppressed by design:

1. *Foreground vs. background:* If 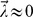, it indicates image background with minor variations due to noise. This case is quantified by 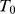, and 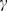 controls the smallest acceptable scale. In membrane locations, 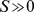 and hence 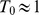. In contrast, background regions have 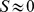 and hence, 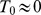.
2. *Plane vs rod:* The parameter 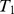 measures the ratio of the largest pair of eigenvalues. It is close to 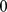 for a plane and 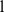 for a rod. Hence, the negative exponential function selectively prefers the plane to a rod.
3. *Plane vs ball:* The term 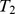 measures the ratio of the smaller pair of eigenvalues with the largest one. It is close to 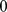 for a plane and in turn, 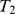 has values closer to 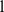. Note that for a ball, 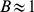 and 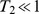 as a result.
4. *Smooth Plane:* In order for 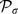 to be a smooth function around the origin (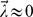) and robust to noise, we add a fourth term 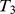 that selectively picks up voxels that have a relatively large 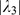 value compared to a small value of 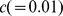. When 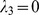, 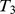 evaluates to 0.

**Figure 3:**
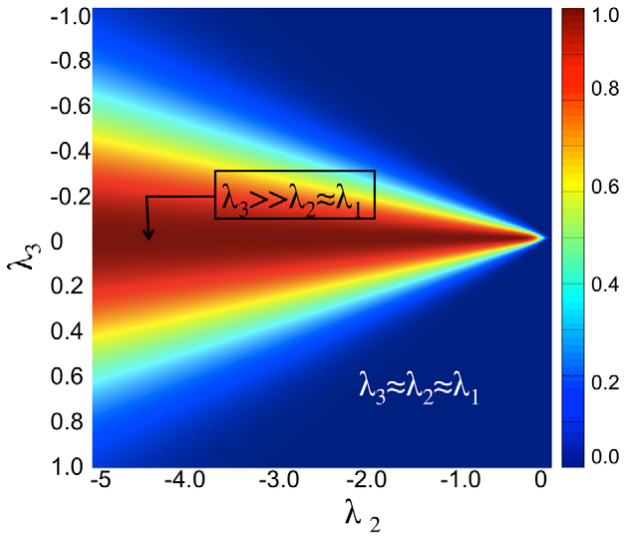
Planarity response function. 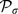
 is computed for different values of (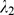, 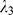) and for 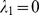 and free parameters 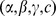 set to 1. The function response shows high values for voxels having 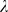 values characteristic of planar arrangements alone.

A heat map of the sampled function 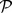 in the 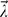 space with all free parameters set to 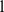 is shown (Figure 3). Since 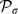 is a three dimensional function defined on (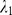, 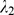, 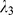), we show a single 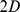 cross-section at 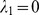. In the figure, high filter response corresponds to voxels having a plane form alone (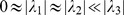), while background voxels and voxels corresponding to the rod and ball forms are suppressed.

Earlier, we described how membranes have crisp or diffuse profiles depending on their orientation with respect to the optical planes (Figure 1). In an ideal situation, the PSF is an impulse-response and the membrane plane is infinitesimally thin and 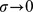 is sufficient for its detection. However, this ideal is not achievable in optical microscopes. So, we model the PSF as an anisotropic Gaussian kernel (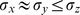). The signal for an orthogonal membrane is contained in a small band of pixels as opposed to an *en-face* membrane which is diffused farther out. Thus, the scale of Hessian computation (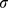) needs to adapt depending on membrane orientation. Let 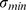 and 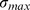 represent the optimal scales for orthogonal and *en-face* membranes with respect to the optical plane with normal 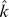 (a unit vector along the optical axis). The normal orientation of membranes locally is given by the eigenvector 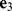, as per our convention. Therefore, we automatically determine scale as:
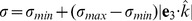
(3)


The dot product of 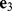 and 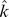 returns the cosine of the angle between the optical axis and the largest principal components, so 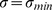 if they are orthogonal and 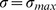 if they are parallel. In our case, for a orthogonal membrane we set 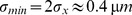 and 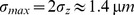, which makes 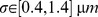. To first determine membrane orientations (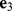 in the above formula), we use a blind scale determined by 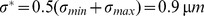 to compute 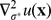.

**Figure 4:**
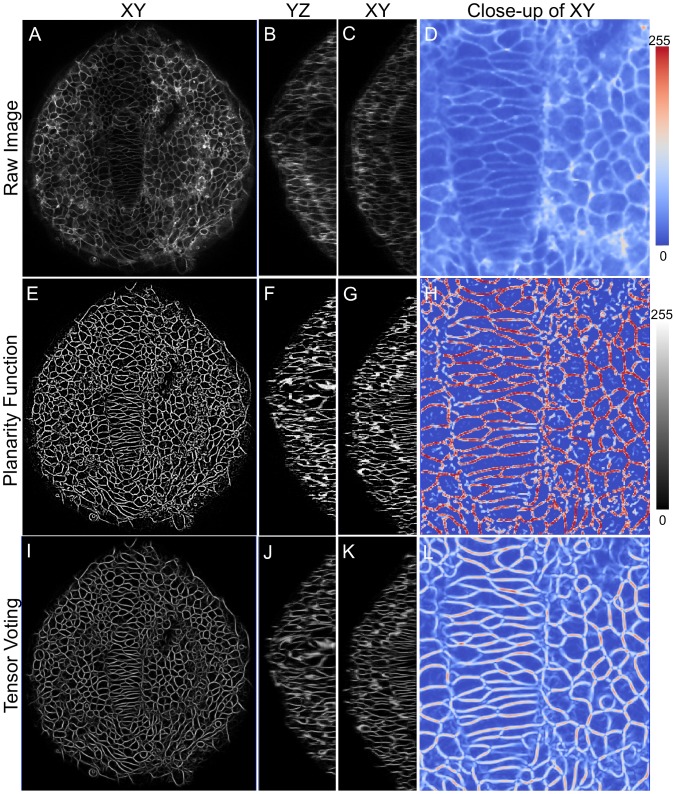
High-fidelity reconstruction of zebrafish membrane images. Significant improvement in membrane signal quality is shown in XY, XZ and YZ planes. (A–D) Raw data showing dorsal view (anterior on top) of zebrafish neuroepithelium (ne) and notochord at 12 hpf, (E–H) Planarity function intermediate output and (I–L) Tensor voting final output. The last image in each panel shows a color-mapped zoomed view for easy comparison.

In Figure 4, we show a three-dimensional result of applying the planarity function on raw data (a–d). The result (e–g) is displayed along orthogonal sections in 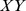, 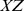 and 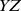 respectively. The last column is a detailed view of the first column of images. We identify membrane voxels inspite of severe intensity inhomogeneities and noise. The image center shows cells in the notochord region which have a very weak intensity profile but were uniformly identified by our method. Local variations of membrane intensities due to orientation differences with the optical planes are also compensated. This can be seen in the orthogonal planes 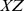 and 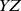, where membrane structures are well-reconstructed. Upon zooming in at a high resolution in (h), we spot several gaps in the membrane structure especially near membrane junctions. At these locations, the intensity structure ceases to be of planar distribution. In some locations, planar noise patterns create false positives in detection. In order to eliminate these spurious structures and reconstruct membranes alone, we use the tensor voting framework to build on the output of the planarity filter.

### Tensor voting to fill structural gaps in membrane data

The principle of tensor voting is that image voxels vote in their surrounding neighborhood to propagate information about the presence of a surface passing through them [21], [22]. At each voxel, votes are cast and accumulated in a local neighborhood. The basic idea behind this process is that if a set of unconnected voxels exists on a geometric surface oblivious of each other, then by voting each voxel develops a sense of direction and affiliation. Thereafter, the surface boundary can be automatically extracted by a region segmentation procedure such as the watershed. The geometric surface in our context refers to the membrane planes.

The application of tensor voting to membrane images has previously been considered. Loss and colleagues developed an iterative extension of the tensor voting framework to demonstrate its application on low fidelity 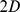 membrane images [23]. Although, tensor voting methods are parameter-free, they are computationally expensive and do not scale well with large image sizes. The iterative extension exacerbates the computational cost. In our case, the accurate detection via the planarity filter provided to the tensor framework eliminates the need for iterative methods. In another extension, Parvin *et al.* [24] developed an iterative voting system that employs tunable kernels to refine paths of low curvature in images. Given the short nature of membrane segments, we do not consider the iterative extension here.

There are three stages of the tensor voting process: (i) initialize a tensor image, (ii) cast and accumulate votes at each voxel, and (iii) extract membrane saliency image.

#### Initialize a tensor image

First, a tensor image 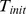 is constructed to represent the affiliation to a geometric surface at each voxel and the corresponding direction of its surface-normal. Each voxel (position 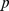) is mathematically represented as a second-order tensor encoding the magnitude as eigenvalues (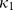, 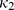, 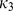) and corresponding eigen directions (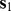, 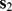, 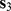). The tensor is a symmetric and non-negative (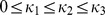) matrix and can be written as:
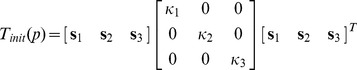
(4)


In a local coordinate system at each voxel, there are three possible geometric structures that can pass through a voxel namely, a 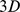 ball, a 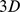 rod, and a 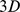 plane. Thus, the tensors encode the contributions of the three forms in terms of their normals as follows:
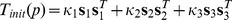
(5)

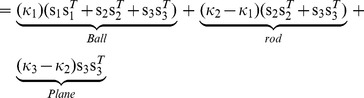
(6)


In the above equation, a plane is encoded as the inner-product of its normal (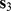), a rod by the inner-product of two normals spanning its cross-section (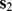 and 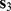), and a ball by the inner-products of all directions (Figure 2). The coefficients 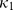, 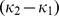, and (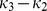) represent the saliency of each geometric structure. At the end of the voting process, we expect membrane voxels to contain high plane saliency 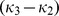 and low saliencies for the rod and ball structures.

To construct a tensor image 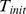, we initialize all voxels as follows: 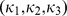 = 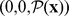 with 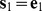, 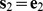, and 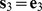. Here, 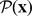 refers to the output of the planarity filter described in the previous section. Therefore, all identified voxels get encoded as plane tensors with high saliencies and directions same as the image Hessian. By substituting in Equation 5, we get the input token image (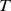) as:
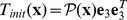
(7)


#### Cast and accumulate votes

Once the initial token image 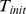 is defined, the next step is the voting step wherein each voxel influences its neighbors in the output image 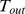 based on a scale parameter 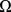. The vote 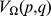 from a voxel 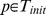 to another voxel 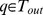 consists of a modified version of the encode tensor 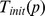. The modification consists of a distance-dependent attenuation of magnitude and transformed orientation of 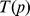. The attenuation in magnitude is motivated by the fact that a voxel's influence should progressively decay in the neighborhood based on its distance from 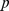. The rotation is motivated by the fact that voting should be cognizant of the curvilinear surface each voxel is affiliated to.

The construction of a plane voting field describing the rotation is given in Supplementary Text S1 (Section 1 and Supplementary Figure S1) and review articles [21], [22]. By using this voting field as a lookup table, the votes from each voxel 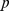 in the input image 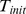 to its neighborhood in the output image 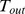 is efficiently computed. There is a single free parameter 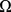 that defines the size of the voting neighborhood window 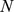.
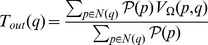
(8)


#### Extract membrane saliency image

We earlier mentioned that the identified voxels in the planarity output belong to either spurious structures generated by noise or lie on 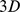 membrane planes. The output of the voting step increases the affiliation (or saliency) of the voxels on the membrane planes and reduces stand-alone or disconnected structures to low saliency values. The output image 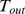 is once again decomposed to its geometric forms using Equation 5 and the plane coefficients 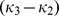 are extracted. This represents the final membrane reconstruction to be used as a topographic map for the watershed algorithm.

Figure 4 shows the reconstructed membrane profile (I–L) given the planarity function input in (E–H). As before, we show the profiles in all three cross-sections of 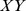, 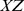, 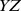 and a zoomed image respectively. It is easy to observe the high quality of reconstruction profiles especially in the zoomed image showing thin and narrow cells. Junctions were smoothly reconstructed and gaps in the structure were eliminated. Spurious formations were also eliminated by the voting process. There is no intensity inhomogeneities present which now make it straight-forward to perform image analysis tasks such as segmentation and shape analysis. We have chosen to focus here on the planar tensor component because our intent is cell membrane segmentation and analysis of morphology, but a similar approach could be used to reconstruct rod-like structures such as microtubules and microfilaments using our code.

### Membrane segmentation using watershed algorithm on reconstructed images

**Figure 5:**
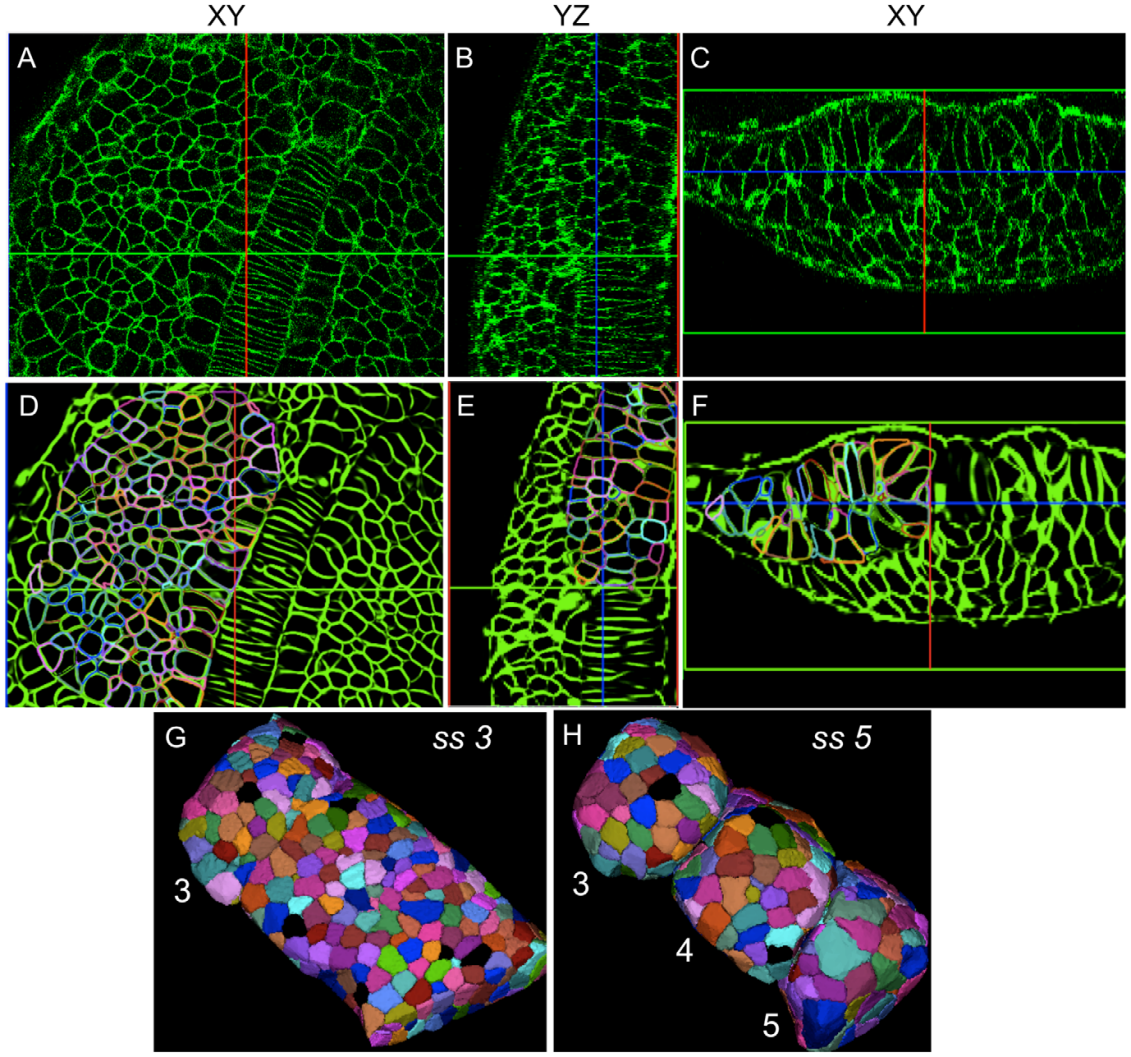
Robust reconstruction and segmentation of cells in the presomitic mesoderm. (A–C) Raw image data showing presomitic mesoderm on 2D image planes (XY,YZ, and XZ) at 3ss. (D–F) Segmentation meshes overlaid on reconstructed membrane images demonstrate excellent localization. Each mesh was randomly colored for visually separating adjacent cells easily. (G,H) 3D rendering of membrane segmentations at 3ss and 5ss. Somites 3, 4 and 5 at 5ss are formed from the presomitic tissue at 3ss by cell sorting and rearrangement.

We use the watershed algorithm for obtaining high quality segmentations once the reconstruction procedure is completed [11]. We use the saliency images generated from tensor voting as topographic maps in the watershed procedure. The saliency image results from the votes of tensors oriented along the membranes thereby causing a very rapid change in values normal to the membrane. Figure 5 shows the resulting cell segmentations obtained from using our new approach on two timepoints of noisy membrane data. The output of the high-quality reconstructed membrane signal is shown in the orthogonal image planes. A step-wise graphical overview of the complete segmentation process is provided in Supplementary Figure S2.

## Results

**Figure 6:**
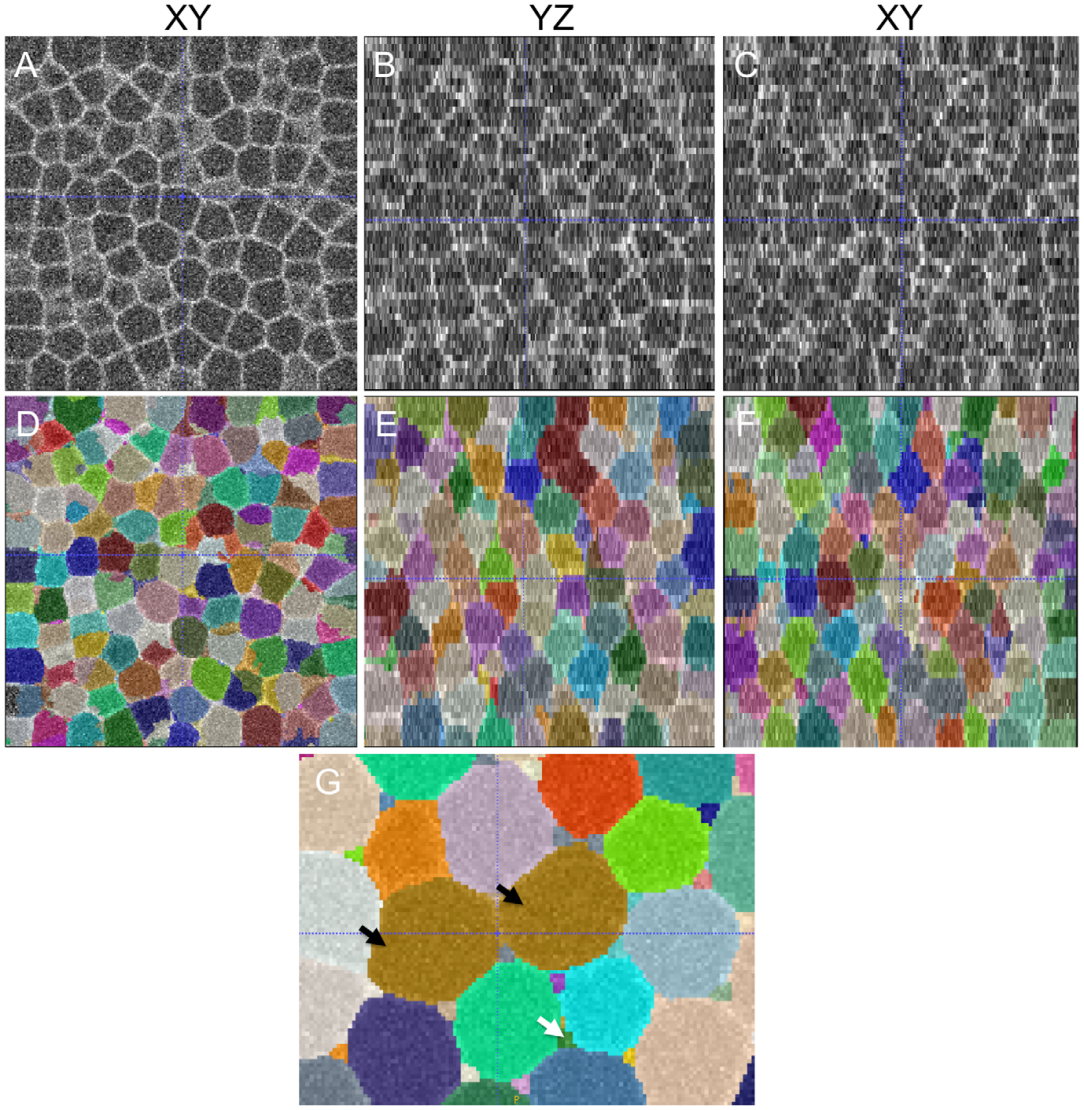
Accurate and highly-sensitive algorithm performance on synthesized 3D membrane images. (A–C) Synthesized cell structures in 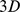 along 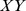, 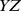 and 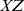 sections with image noise added (Table 2). As in the case of real-world images, the lateral resolution significantly differs from the axial resolution. (D–F) Segmentations overlaid on the raw image with a 50% opacity function. (G) An example of under-segmentation (brown cells, black arrows) and over-segmentation (interstitial fragments, white arrows) in the image. The errors could be filtered out by size criteria.

In order to validate our segmentation results, we quantified segmentation accuracy on synthetic images where ground truth is known and on real images manually segmented by experts using four metrics: average volume overlap (Dice), average L2 Hausdorff distance, over-segmentation and under-segmentation rates. The Dice coefficient for measuring volume overlap between the automated results and the ground truth for a single cell is defined as:
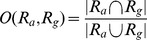
(9)where 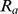 is the automated extracted region and 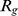 is the ground truth region. The 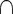 operator takes the intersection of two regions while 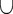 takes the union of regions. The L2 Hausdorff distance (in 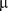
m) refers to farthest separation of closest boundary points between the two segmentations [25]. In other words, it is the error in localizing the true border between two cells due to distortions in the object morphology. The over-segmentation measure indicates that a cell has been separated into more than one object, or an extracted object has not been labeled as cell. The under-segmentation measure indicates that clusters of cell have not been appropriately divided or a true cell was not at all extracted (Figure 6G).

In Tables 2 and 3, a total of 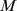 pairs of manual (of total of 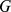 labels) and automated segmentation labels (of total of 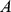 labels) were first matched by checking for overlap larger than 0.75. The average volume overlap (Overlap) and L2 Hausdorff metric (Encroach) was computed across all 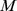 matched pairs. Manual segmentation labels that remained unmatched were classied as over-segmentation (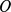) or under-segmentation (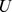) labels. We define an over-segmentation instance when a manual label overlaps with multiple automated labels. Under-segmentation is when two manual labels are output as a single automated label. However, there is a scope for complex error types to be present. For example, an automated label may undersegment two manual labels but participate in the over-segmentation of a different manual label. To be consistent, over-segmentation is when a manual label has no more than 75% of its area overlapping with any automated label (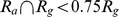), else it is an under-segmentation label. Based on these classifications, we measured the precision and recall of the automated procedure. Precision measures the fraction of correctly identified cells from the automatically segmented set of cells (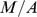), so measures false negatives. Recall measures the fraction of correctly identified cells from the number of manually segmented cells (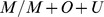) so measures false positives.

**Table 2:** High sensitivity in algorithm performance on synthetic data with varying noise parameters.

Data	( 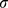 , 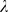 )	#Cells	U	O	M	Dice	Encroach	Prec.	Recall
1	(0.01, 1.0)	1000	0	0	1000	0.99	0.25	1.0	1.0
2	(0.02, 0.9)	1000	0	0	1000	0.97	0.27	1.0	1.0
3	(0.03, 0.8)	1000	0	0	1000	0.94	0.35	1.0	1.0
4	(0.04, 0.7)	1005	0	5	1000	0.92	0.47	0.99	1.0
5	(0.05, 0.6)	1010	2	12	998	0.91	0.52	0.98	0.99
6	(0.06, 0.5)	1021	4	25	996	0.89	0.70	0.97	0.99
7	(0.07, 0.4)	1027	6	33	994	0.87	0.85	0.96	0.99
8	(0.08, 0.3)	1032	8	40	992	0.87	1.11	0.96	0.99
9	(0.09, 0.2)	1033	11	44	989	0.86	1.31	0.95	0.98
10	(0.1, 0.1)	1038	16	54	984	0.84	1.42	0.94	0.98

Algorithm performance was measured against ten synthetic datasets with progressively higher noise parameters (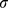,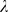) and increasing cell number. **Dice** refers to the area of overlap between ground-truth and automated segmentations. **U**, **O**, and **M** list the number of cells that were undersegmented, oversegmented, and matched respectively. **Encroachment** measures the average L2 Hausdorff displacement of the cell boundaries (a lower value is better). **Precision** measures the fraction of correctly identified cells from the automatically segmented set of cells. **Recall** measures the fraction of correctly identified cells from the manually segmented set of cells. The algorithm recorded a precision of at least 94%, recall of at least 98%, and an volume overlap of at least 84% even with high levels of noise, thereby indicating an accurate performance of the method.

**Table 3:** Robust correspondence of automated segmentations with manually segmented zebrafish membrane images.

Dataset	#Cells	Algorithm	O	U	M	Dice	Encroach	Precision	Recall
		1	6	9	37	0.88	0.45	0.63	0.71
1	52	2	3	4	45	0.91	0.39	0.81	0.86
		3	2	2	48	0.93	0.21	0.88	0.92
		1	8	7	43	0.90	0.37	0.65	0.74
2	58	2	4	3	51	0.92	0.41	0.82	0.87
		3	4	2	53	0.94	0.28	0.89	0.91
		1	7	8	47	0.87	0.52	0.68	0.75
3	62	2	4	3	55	0.90	0.47	0.83	0.88
		3	2	2	58	0.91	0.31	0.90	0.93
		1	10	12	42	0.91	0.42	0.56	0.65
4	64	2	7	5	52	0.91	0.41	0.73	0.81
		3	3	2	59	0.95	0.29	0.88	0.92
			13.06%	13.48%		0.89	0.44	0.63	0.71
Average			7.51%	5.92%		0.91	0.42	0.79	0.85
			4.66%	3.3%		0.93	0.27	0.89	0.92

Automated algorithm performance was measured against manually-segmented membrane images of the zebrafish presomitic mesoderm from four different time-points. The proposed algorithm 3 recorded an average precision of 89%, average recall of 92%, average encroachment of 0.27 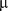
m, and average volume overlap of 93%, thereby indicating an accurate performance of the method. Moreover, algorithm 3 consistently performed better than the basic watershed procedures (algorithms 1 and 2) across all the chosen metrics indicating the utility of the reconstruction procedure in improving algorithm performance.

### High sensitivity as demonstrated by performance on synthesized 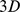 membrane images

Since there is no gold standard available for evaluating algorithm performance, we synthesized 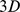 membrane images based on an image formation model that simulates confocal microscopy of membrane labeled embryos (Supplementary Section S3 and Supplementary Figure S3). The advantage of using synthetic images is that ground-truth is exactly known and different imaging parameters can be rapidly tested. A spectrum of ten images with varying noise parameters was generated for comparison, and the performance of the algorithm is described in Table 2. Despite the fact that cells are tightly-packed and large additive noise is present, the proposed method reconstructs membranes and segments the touching cells with high precision. Figure 6(A–C) shows an example of a synthesized 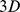 membrane image containing 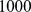 cells with the corresponding segmentation results shown in Figure 6(D–F). The performance of the algorithm steadily degraded for higher levels of noise as expected. It was observed that in the worst case, we obtained a precision of 94% and recall of 98%. The enchroachment on neighboring cells was limited to 1.42 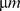 and with an overlap of more than 84%. As it is clear from these results, our proposed segmentation method achieves significant volume overlap with the ground truth, indicating the accurate performance of the segmentation method.

### High sensitivity and segmentation accuracy as demonstrated by performance on manually-segmented zebrafish membrane images

We next applied the method to images of zebrafish mesoderm obtained at 12 hpf (Figure 4). Four 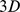 membrane images were used to evaluate the proposed segmentation method. Using the publicly available GoFigure2 software, an expert manually marked all the somite cells in a small image region by drawing 2D contours on different image planes. For each cell, a 3D mesh was generated out of the sampled 2D contours by using an automatic surface reconstruction procedure. The 3D meshes were used to compare and assess the performance of the automated segmentation algorithm. To demonstrate the effectiveness of our reconstruction procedure for automated segmentation, we compare the performance of three versions of the automated algorithm, namely:

1. watershed on intensity data directly,
2. watershed on planarity filtered data, and
3. watershed on planarity filtering and tensor voting.

In Table 3, we evaluated the segmentation metrics and observed that the basic algorithms 1 and 2 suffer from a high over-segmentation (13.06%, 7.51%) and under-segmentation (13.48%, 5.92%) error rates. Over-segmentation occurs when spurious structures are present in cell interiors that split single cells into multiple labels. Under-segmentation rates are high due the lack of membrane pixel connectivity especially in 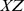 and 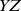 planes. When segmentations correctly matched, the algorithms localized the boundary accurately (0.44 and 0.42 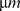) and also had a significant volume overlap (89% and 91%). In contrast, algorithm 3 shows significantly improved performance. On average, the over-segmentation and under-segmentation rates are 4.66% and 3.3% respectively. For the matched set of cells, the average volume overlap and L2 Hausdorff distance are 93% and 0.27 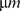, respectively demonstrating the low distortion in object morphology. Our results indicate that the reconstruction procedure enhanced membrane connectivity and eliminates spurious structures, thereby reducing the over and under-segmentation error-rates.

### Robust performance as demonstrated by an exploration of the scale space parameters

**Figure 7:**
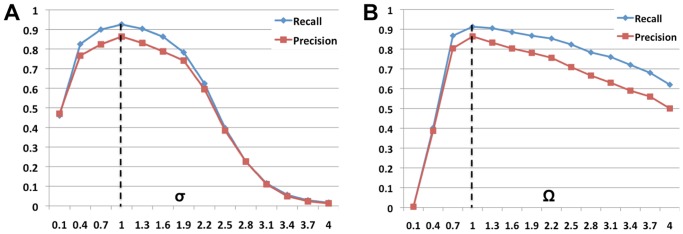
Scale exploration demonstrates robust algorithm performance. Precision and recall measures are plotted against different settings of (A) 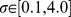, 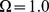 and (B) 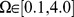, 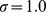. Precision and recall values were maximized with 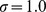 and 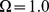 and and gradually decreased over broad range of parameter settings indicating robustness. Low scale settings generated noisy features leading to higher over-segmentation rates while large scale settings tended to smooth out sharp membrane corners and cause under-segmentation errors.

The two scale parameters 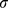 and 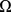 constitute two important parameters in generating the automated output. Thus, we explored a range of 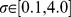 and 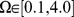 values and assessed the variation in the performance of our method. While changing a given parameter, we ensured the other parameters were optimally set. Figure 7 reports the average precision and recall values plotted against 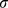/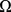 values for the four manually segmented datasets. We observe robust performance for a broad range of 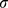 and 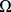 parameters ([0.7, 1.5]) with a gradual degradation in performance. High values (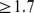) tend to assign more importance to membrane smoothness over large scales. This negatively impacts membrane connectivity at cell junctions leading to under-segmentation and hence lowers the precision/recall metrics. Low values (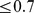) tend to localize membranes more accurately but retain spurious structures that causes over-segmentation and also lowers the metrics. Hence, a judicious choice that balances over-segmentation and under-segmentation rates is recommended.

### Robust correspondence between membrane and nuclear segmentations

**Figure 8:**
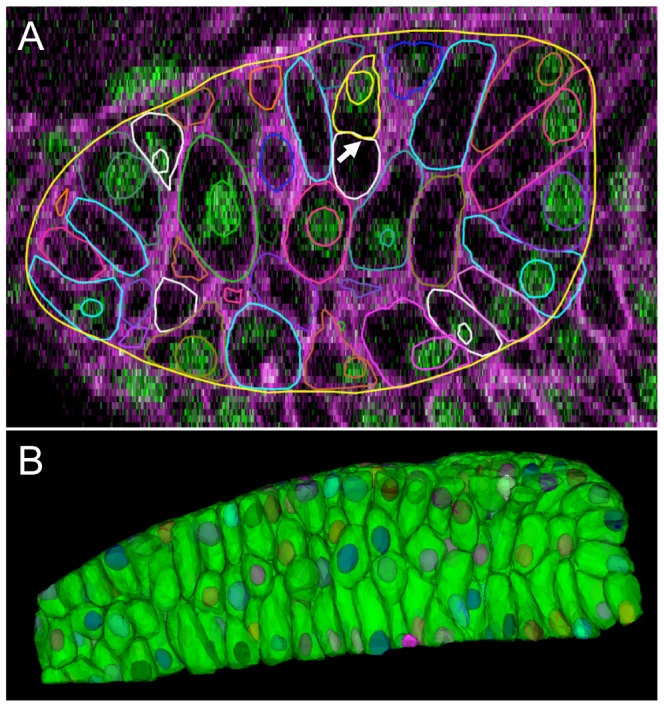
Robust correspondence between membrane and nuclear segmentations. Algorithm performance was assessed by matching automated segmentations obtained from the nuclear and membrane channels. In the ideal case, each individual nucleus would match with a unique membrane and vice-versa. (A) A single 2D image plane is shown with contours of membrane and nuclear segmentations overlaid on raw data. Some cells have their corresponding nuclei located out-of-plane. The lack of a one-to-one correspondence indicates an error. For example, an over-segmentation of the membrane channel (white arrow) causes one of the membrane components to not contain a nucleus. (B) 3D renderings of cells from membrane and nuclear segmentations.

We also applied the method to three 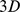 zebrafish images in which nuclei and membranes are imaged in separate channels and segmented separately. For nuclear segmentation, we use an improved version of the watershed algorithm using seeds [26]. Although the nuclear and membrane segmentations are not perfect, in an ideal scenario there should exist a one-to-one correspondence between both segmentations (Figure 8). We extract the centroids of cells and nuclei and match them using a nearest neighbor method. Table 4 provides the details of the matching. On average, the number of nuclei extracted are less than the number of cells from membrane information. This discrepancy is because interstitial space in the tissue (or vacuoles) can be segmented as cells even when they do not exist. These empty spaces can be difficult even for a human to distinguish in the absence of any other information. Our experiments demonstrated an excellent match between the nuclear and membrane segmentation algorithm outcomes indicating a robust performance of our segmentation software.

**Table 4:** Robust correspondence of automated membrane segmentations with automated nuclear segmentations.

Data	#Cells	#Nuclei	#Matched	#Unmatched Cells	#Unmatched Nuclei
1	312	291	279	33	12
2	217	194	186	31	8
3	241	228	219	22	9

Detection and error rates of the automated algorithm was compared with standard nuclear segmentation algorithms. The assumption was that perfect segmentations of both algorithms should theoretically establish a one-to-one correspondence between nuclei and membranes detected. **Matched** refers to cells with membrane and nuclei in exact correspondence. **Unmatched Cells** refer to membranes that did not contain a unique nucleus. **Unmatched Nuclei** refer to nuclei that did not correspond to a cell membrane.

### Quantitative analysis of cell shape and size during somite formation

During zebrafish somitogenesis, a series of epithelial tissue blocks forms rhythmically by separating from the presomitic mesoderm tissue (PSM) [27]. A total of 28 pairs of blocks known as somites sequentially form beginning at 10 hpf with a period of approximately 30 minutes [28]. Somites are formed by cell sorting from the PSM. Each somite is structurally composed of epithelial (E) cells on the boundary with an inner mesenchymal (M) core. Throughout somite formation, the PSM maintains a steady-state by coordinating the anterior process of somite formation with cell recruitment and proliferation at its posterior end. The PSM is gradually patterned along the anteroposterior axis by cellular rearrangements and tissue/cell-shape changes, deriving its input from an oscillating molecular circuit known as the *segmentation clock* (not to be confused with image segmentation) [29], [30]. Segmentation clocks operating inside individual cells are synchronised along the PSM to create periodic waves of oscillating gene expression. While there has been substantial progress in understanding the molecular mechanisms of wave initiation, synchronization, and the readout circuitry, the cellular and mechanical mechanisms involved in physically sculpting a somite are not clear due to the lack of high-quality image data and subsequent robust analysis [31]. For example, it is not exactly known how the sorting interface develops, what the cell movement patterns at the interface are, how many cells are involved, and what the corresponding changes in cell and tissue morphology are.

**Figure 9:**
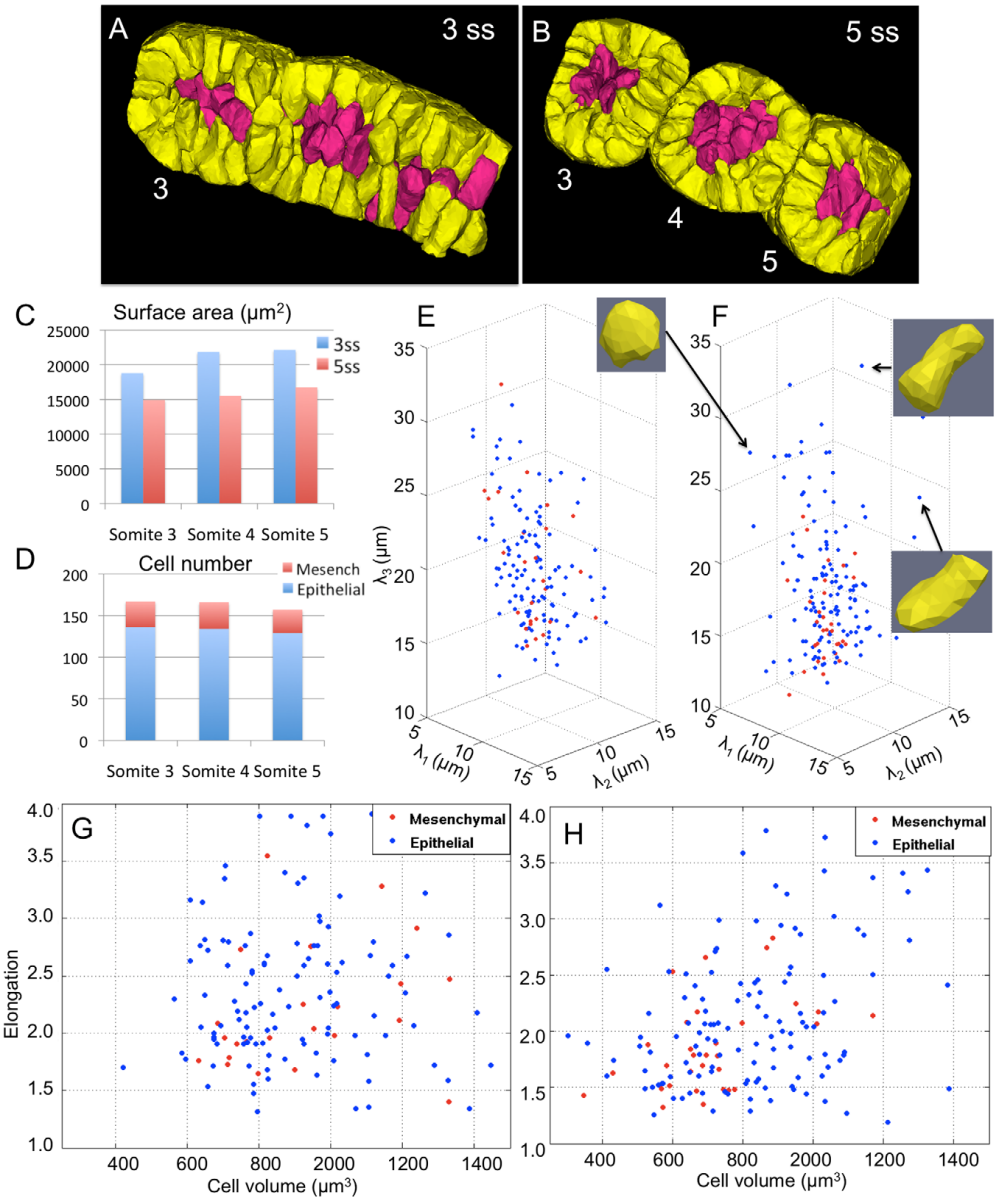
Algorithm-enabled quantification of cell dynamics during somite formation. Retrospective cell tracing of epithelial (yellow) and mesenchymal (red) cells from formed somites at (B) 5ss back to the presomitic mesoderm at (A) 3ss. (C) Corresponding decrease in somite tissue surface area during the formation of somites 3, 4, and 5. (D) Epithelial and mesenchymal cell numbers in respective somites at 5ss. (E,F) Three-dimensional cell shape quantified by the length of their principal axes at 3ss and 5ss. (G,H) Scatter plots of elongation (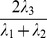) and cell volumes at 3ss and 5ss. The two cell populations show different behavior. Statistical analysis of the two distributions show that mesenchymal cells (red) tend to cluster, round-up, and shrink in size on average.

Therefore, our goal was to obtain time-lapse membrane images during somite formation, apply our reconstruction techniques, and quantify cell dynamics. We chose to *in toto* image the formation of somites 3, 4, and 5 in a zebrafish embryo mounted dorsally, with a 40× objective, and with a time-sampling of 2 minutes over a period of 60 minutes using confocal microscopy [32]. The beginning marked the formation of somite 3 with a discernable interface with the presomitic mesoderm. During the time-lapse, we observe the complete separation of somite 3 and 4. Somite 5 forms a discernable interface at the end of the time-lapse thus completing two full cycles of segmentation. In Figure 5, we present the results of our reconstruction (orthogonal sections) and automated segmentation (3D view) of the PSM cells at 3 and 5 somite stages (ss). Automated segmentations were overlaid on the reconstructions to show the excellent agreement in contours. We then proceeded to analyze the formation of somites by quantifying differences between 3 and 5 ss (Figure 9). As a consequence of somites physically separating and becoming spherical, interface surface area decreases across all the three somites. Given that somite 3 is farther along than somite 4 and somite 5 in the process of forming round somites, their surface areas (see blue and red bars) are monotonically higher. In particular, somites 4 and 5 show a large surface area in the PSM initially (see blue peaks). At ss 5, somite 3 and 4 show significantly smaller surface area due to the completion of somite rounding while somite 5 is still halfway through. Thus, the blue peak at somite 3 roughly corresponds to the red peak of somite 5 given the same relative progress into somite formation. The total number of cells is very consistent across the three somites (Figure 9D).

In order to understand the corresponding changes in cell parameters, we then identified the number of epithelial and mesenchymal cells in the formed somites. Mesenchymal cells which do not touch the surface of the somite were a small fraction (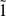
5–20%) of the cells (Figure 9D). The epithelial layer formed a single layer of cells over the mesenchymal cells. We labeled these cells in different colors and retrospectively tracked their location into the PSM. For tracking, segmentations at individual time-points were linked based on optical flow-fields that were first reconstructed [33]. Errors in the tracking were corrected manually using the publicly-available GoFigure2 software (www.gofigure2.org). We then computed the principal diameters of the cells along their principal axis. A typical workflow consists of first visualizing and interactively exploring the distributions of cell shapes, sizes, and locations after the segmentation process is completed. The process of interaction involves zooming in and out, changing the viewing angle, hiding a subset of data, and visualizing specific outliers or data points. Scatterplots in Figure 9(E–F) show the distributions of the epithelial (blue) and mesenchymal cells (red). In ss 3, we observe large and homogenous variation of cell morphology across E and M classes. At ss 5, M cells form a more narrow distribution while E cells spread out to form diverse cell shapes neccessary for constructing spherical somites with continuous epithelia. A few examples of such cells are shown in (F). After finding interesting correlations and trends, accurate 2D scatterplots or figures can be used for effective visualization. Hence, we also computed changes in cell volumes (size) in Figure 9(G–H). Here, we observe that the distribution of mesenchymal cell volumes (red) narrows and interestingly we find that the mesenchymal cells shrink in volume as the somite forms. We are now analyzing somite formation rigorously across all intermediate time-points, combined with tracking results for individual cells and across multiple datasets. As part of our future work, we plan to integrate the process with the underlying molecular circuitry to obtain a multiscale view of somite formation.

Our work successfully demonstrates the utility of our algorithms in enabling the quantification of cell shape and size, tissue interface areas and volumes, and reconstruction of cell lineages and fate maps by tracking segmented cells. By recovering individual cell dynamics and their collective behavior in tissue from time-lapse images, a deeper understanding of the mechanisms involved in morphogenesis can be obtained. Thus, our algorithms are computationally robust and can be deployed to facilitate the analysis of a wide-variety of morphogenesis systems.

## Availability and Future Directions

Our method has several advantages over previous approaches. The first major advantage of the method is the ability to robustly segment tightly-packed cells without relying on their absolute fluorescence levels. Since we detect membranes based on local shape information computed from second derivatives of the image intensity function, the absolute values are not important. This is very relevant for time-lapse imaging data because membrane-tagged fluorophores can photobleach. With our method, it will be possible to segment cells and track them for a longer developmental time-window using only the membrane channel. The second major advantage is that our technique deals with intensity inhomogeneities that occur in membrane surfaces due to their orientation with respect to the imaging planes. Our method can easily be extended to using nuclear information when available as seed-points for the watershed that will further reduce the amount of over and under-segmentations. Conversely, the reconstructed and localized membranes can also be used to refine nuclear segmentations. Currently the method is implemented in C/C++ language and uses The Insight Toolkit (ITK) libraries (http://www.itk.org/) that are open-source and publicly available. We have used multi-threading optimization strategies and efficient data structures to take in account modern multicore computer architectures. Our software can be readily used in a cluster environment for large scale image processing. The documentation provided with the source code (see Supplementary Text S1, Section 3) details the set of steps required to download, compile, link, and execute the code. Our software has been developed and tested on Windows, Mac, and Linux platforms and is available publicly under a BSD license (https://github.com/krm15/ACME/). A copy of the source code and scripts used in the preparation of this manuscript is provided as a zipped file in Protocol S1. Precompiled binaries are also available at https://wiki.med.harvard.edu/SysBio/Megason/ACME. A single time-point of our somite image data (Dataset S1) used in the paper and all of our synthetic data (Dataset S2) are provided. Default parameter values are provided as well as instructions for modifying them, if needed. Code for generating new synthetic data with other parameter values has also been included in the repository.

In conclusion, our software enables the efficient and accurate quantification of cell shape, size, and position from large time-lapse images in an automated manner. We believe that this work is immensely useful to research aimed at understanding individual and collective cell behavior using high-resolution microscopy, especially in the context of tissue morphogenesis and organ formation.

## Supporting Information

Dataset S1 **A single time-point of somite image data along with intermediate processing results from using ACME code.**
(ZIP)

Dataset S2
**Ten synthetic image sets generated with progressively higher noise parameters (** 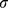 **,** 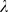 **) and increasing cell number.**
(ZIP)

Figure S1 **Tensor voting field determination.** (A) 2D voting field parameters. (B) Heat map showing the stick voting field saliencies in 2D. The stick tensor is represented using line glyphs and overlaid on the figure. (C) A simple example showing two sampled intersecting circles and their reconstruction (D).
(TIF)

Figure S2 **A flowchart of processing filters and parameters with intermediate outputs.** There are four filters that take the input image to produce an output segmented image. For each step on the left, the corresponding input and output image is shown on the right.
(TIF)

Figure S3
**Synthetic membrane images along XY, XZ, and YZ sections.** The (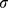, 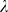) values were sampled as (A–C) (0.01, 1.00), (D–F) (0.05, 0.6), and (G–I) (0.1, 0.1). Corresponding ground truth segmentation images (XY) are shown in (J–L).(TIF)

Protocol S1 **A compressed zipped file containing ACME source code in C++, a README file, and python scripts for generating synthetic image data.**
(ZIP)

Text S1 **Step-by-step instructions for downloading, compiling, and executing ACME code on provided test datasets.**
(PDF)
