# Supplementary figures and images for: ACME: Automated Cell Morphology Extractor for Comprehensive Reconstruction of Cell Membranes
Source: PLoS Comput Biol. 2012 Dec 6;8(12):e1002780. doi: 10.1371/journal.pcbi.1002780 (PMC3516542; doi:10.1371/journal.pcbi.1002780)

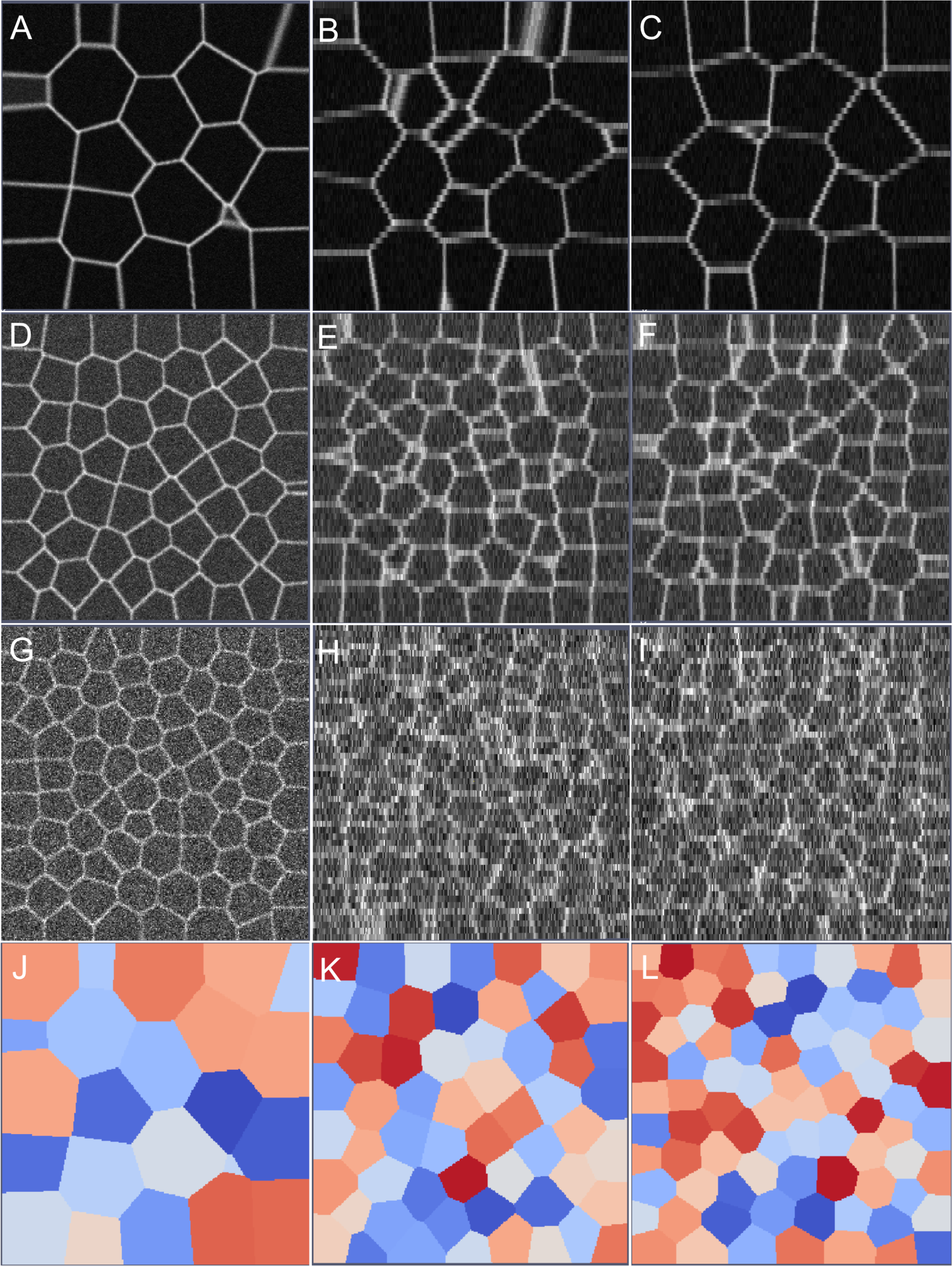

Supplement: Figure S1 — Tensor voting field determination. (A) 2D voting field parameters. (B) Heat map showing the stick voting field saliencies in 2D. The stick tensor is represented using line glyphs and overlaid on the figure. (C) A simple example showing two sampled intersecting circles and their reconstruction (D). (TIF) [file pcbi.1002780.s003.tif]

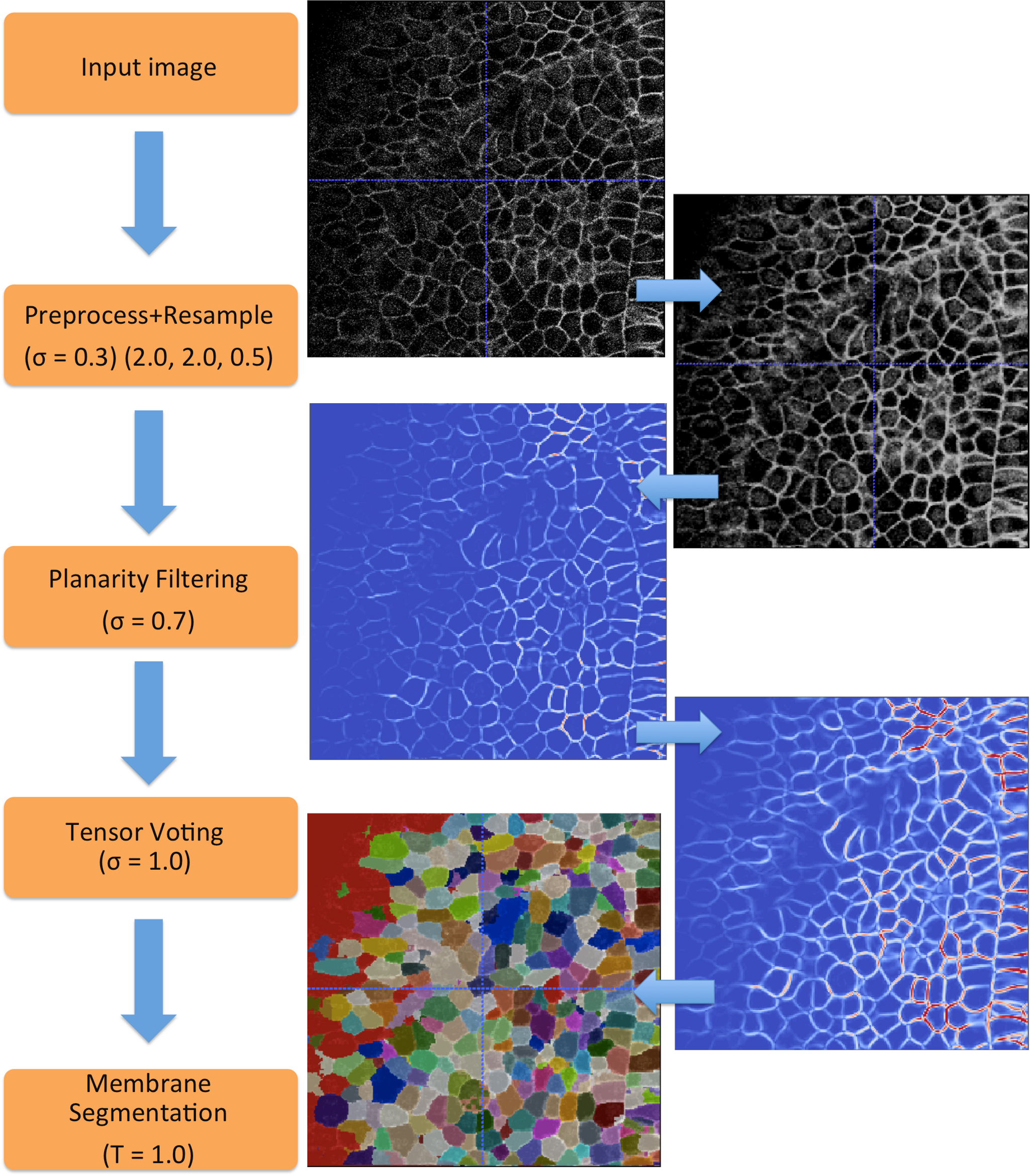

Supplement: Figure S2 — A flowchart of processing filters and parameters with intermediate outputs. There are four filters that take the input image to produce an output segmented image. For each step on the left, the corresponding input and output image is shown on the right. (TIF) [file pcbi.1002780.s004.tif]

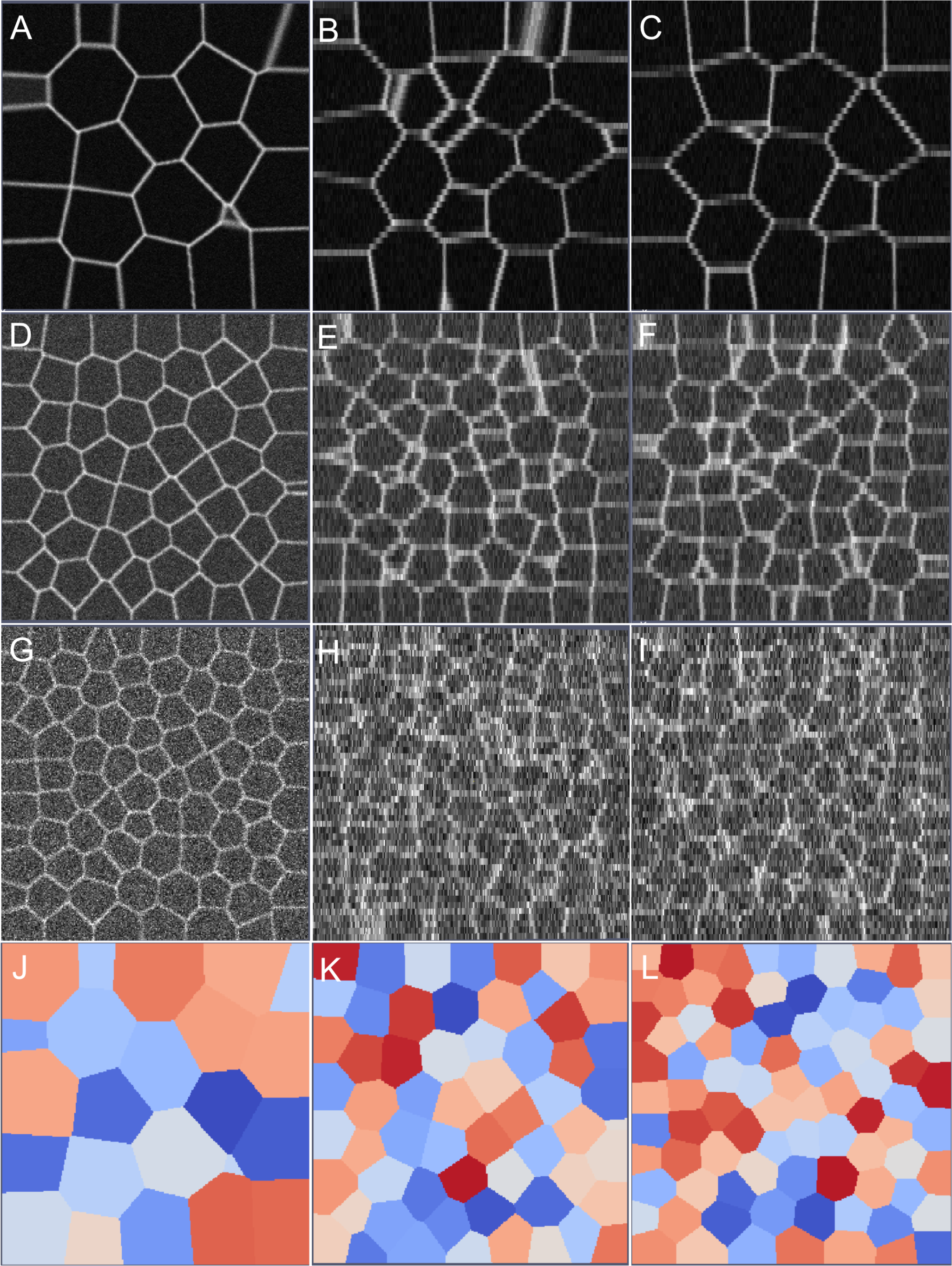

Supplement: Figure S3 — Synthetic membrane images along XY, XZ, and YZ sections. The (, ) values were sampled as (A–C) (0.01, 1.00), (D–F) (0.05, 0.6), and (G–I) (0.1, 0.1). Corresponding ground truth segmentation images (XY) are shown in (J–L). (TIF) [file pcbi.1002780.s005.tif]
