# Supplementary material for: ACME: Automated Cell Morphology Extractor for Comprehensive Reconstruction of Cell Membranes
Source: PLoS Comput Biol. 2012 Dec 6;8(12):e1002780. doi: 10.1371/journal.pcbi.1002780 (PMC3516542; doi:10.1371/journal.pcbi.1002780)
Supplement: Text S1 — Step-by-step instructions for downloading, compiling, and executing ACME code on provided test datasets. (PDF) [file pcbi.1002780.s007.pdf]

Supplementary Information

1 Tensor voting fields

Consider a plane tensor with normal having unit magnitude (saliency) and aligned with Y-axis as shown in Figure S1(a). Intuitively, this indicates a curve element (surface in $3D$) with normal along Y-axis. Now, consider the orientation and saliency of the curve to pass through any other point p. Using the Gestalt principles of perception in human vision systems, continuity and proximity are essential in making a determination [2]. For a given position $p = (x, y)$ and at an angle θ, the vote V at p, is determined as:

$$V^{2D}(p) = \exp \frac{s^2 + c\kappa^2}{\sigma^2} NN^T \tag{1}$$

Here, s is the arc length and κ is the curvature of a smooth osculating circle passing through p. N is the normal vector at p to the smoothest path between the two tensors and is given by $[sin(2\theta) \quad cos(2\theta)]^T$. In Figure S1(b), the $2D$ plane voting field is shown. The heat map has a range of $[0, 1]$ and the tensor orientations are overlaid at discrete locations. Note that the plane field exist only at $\theta \leq 45$ and tensor saliencies attenuate with increasing distance and curvature of the perceptual structure. A plane tensor field V_{3D} in $3D$ is obtained by rotating the $2D$ voting field obtained about the Y-axis which is the axis of symmetry. Given Euler angle transformations (θ, ϕ, ψ) about X, Y and Z-axis respectively, we can align the voting fields as:

$$V^{3D}_{\theta\phi\psi} = R_{\theta\phi\psi} S^{3D} R^T_{\theta\phi\psi} \tag{2}$$

Figure S1(c) shows a simple example in which voxels are drawn from two intersecting circles. Each voxel is initialized as a plane token of unit saliency with e_1 along the normal. The reconstruction shows the extracted plane saliency map with correct normal orientations.

2 Software Usage

For the convenience of users, we have provided 32- and 64-bit precompiled binaries for Windows, Mac, and Linux environments at *https://wiki.med.harvard.edu/SysBio/Megason/ACME*

Users can download the appropriate binaries, unzip, and follow the commands from Supplementary Section 2.3 onwards. For advanced users, we list the commands to directly download source code, compile and use our software for membrane processing. We assume that the computing environment is Linux here but similar steps can be listed out easily for the Mac and Windows environments. The software has been designed and tested in a cross-platform environment. We use CMake, a tool that generates makefiles under Linux, the Visual Studio Solution files (.sln) under Windows, or the XCode project files (.xcodeproj) under Mac OS X. It allows developers to build their projects across different platforms with a minimum number of reconfiguration steps. We use GIT, a version control system used to manage our source code. The GIT repository is located at *https://github.com/krm15/ACME/*

Our software uses the Insight Toolkit (ITK) library (*www.itk.org*). ITK is an open-source, cross-platform system that provides developers with an extensive suite of software tools for image analysis. In what follows, statements beginning with a $ symbol refer to commands to be executed in a terminal window.

2.1 Downloading and compiling ITK

1. Download ITK to a directory called ITK-Source
$ mkdir ITK-Source

$ cd ITK-Source
$ git clone git://itk.org/ITK.git
$ cd ITK
2. Fetch any submodules in the ITK tree:
$ git submodule update –init
3. Create build directory for compiling ITK code. We refer to this directory as ITK-Binary.
$ mkdir ITK-Binary
$ cd ITK-Binary
4. Configure ITK. Turn on the variables ITK_BUILD_ALL_MODULES, ITK_USE_REVIEW and set CMAKE_BUILD_TYPE to Release.
$ ccmake <PathToITK-Source>/TK-Source
5. Build ITK
$ make

2.2 Downloading and compiling ACME software

6. Download software to a directory called MR-Source
$ mkdir MR-Source
$ cd MR-Source
$ git clone https://github.com/krm15/ACME.git
$ cd ACME
7. Create build directory for compiling the software. We refer to this directory as MR-Binary.
$ mkdir MR-Binary
$ cd MR-Binary
8. Configure the software build. It is important to set CMAKE_BUILD_TYPE to Release and enter the path to the ITK build directory (ITK-Binary) from step 3.
$ ccmake <PathToMR-Source>/MR-Source
9. Build the software
$ make

2.3 Running the software on a 2D intersecting circle voting example

8. The source code for this simple test is located in
MR-Source/Examples/BallVotingIntersectingCirclesExample2D.cxx. There are 3 inputs to set in the command line. The voting radius *sigma* and the names of two output images showing the tokens and the reconstructed circle. For simplicity, we use the flexible MHA image format that is capable of storing multi-dimensional ($2D/3D$) data with any datatype (int/float/double/vector/tensor). To run the example, we go to the binary directory to execute the command.
$ cd MR-Binary
$ bin/ballVotingIntersectingCirclesExample2D 1.0 Data/BallVotingExample/inputTokenImage.mha Data/BallVotingExample/outputReconstructedImage.mha
The image inputTokenImage.mha is the set of automatically generated points from two intersecting circles. The output image outputReconstructedImage.mha is a vector image with three components indicating the presence of three structures. We have shown the stick component in Figure S1(b). Upon exploring the other components, the reader can also observe the exact points where the circles intersect.

2.4 Running the code on somite data

Here, we start with the processing of a single timepoint of somite data. Our input raw data is availabe for download as supplementary files (Dataset S1). (*DatasetS1/PresomiticMesoderm/Somite0.mha*). We sequentially run four filters for preprocessing+resampling, planarity filtering, tensor voting, and watershed segmentation to obtain a reconstructed membrane image shown in Figure S3. The code for the planarity filter and tensor voting classes are located in *MR-Source/Code/PlanarityFilter/ MR-Source/Code/TensorVoting/* respectively. All the example code described below for running the filters is located in the folder *MR-Source/Code/Examples/.* The compiled code for executing the commands should be located in *MR-Binary/bin.* In Figure S2, we provide a flowchart of the processing steps, the flow of information, and the corresponding intermediate outputs at each stage.

9. Preprocess the images to eliminate photon shot-noise during acquisition - This is a simple median filter with hole-filling. Source code for this step is located in *MR-Source/Code/Examples/CellPreprocess.cxx.* There are three command-line parameters namely, the input image, the denoised output image, and the average cross-sectional radius of a membrane in physical units approximately. The average radius in our images was set to 0.3 μm.

$ ./cellPreprocess DatasetS1/PresomiticMesoderm/Somite0.mha
DatasetS1/PresomiticMesoderm/Somite0-preprocess.mha 0.3

10. Resample the images to make the voxel sampling isotropic - While this step is not required for the normal operation of the reconstruction code, it is required for the optimal operation of the watershed segmentation algorithm. This is a simple linear interpolation that changes the image sampling from 0.2:0.2:1.0 to 0.4:0.4:0.5. Source code for this step is located in *MR-Source/Code/Examples/Resample.cxx.* There are five command-line parameters namely, the input image, the resampled output image, and resampling mutliplication factors.

$ ./resample DatasetS1/PresomiticMesoderm/Somite0-preprocess.mha
DatasetS1/PresomiticMesoderm/Somite0-preprocess.mha 2.0 2.0 0.5

11. Apply the planarity detection filter - The source code for this filter is located at *MR-Source/Code/Examples/MultiscalePlateMeasureImageFilter.cxx.* The planarity filter expects four parameters - the preprocessing input image, the planarity filter output image, the eigen matrix image, and the neighborhood size for locating planar structures in physical units. Please note that the eigen matrix image stores a 3×3 matrix at each pixel and hence uses a relatively high amount of memory.

$ ./multiscalePlateMeasureImageFilter DatasetS1/PresomiticMesoderm/Somite0-preprocess.mha
DatasetS1/PresomiticMesoderm/Somite0-planarity.mha DatasetS1/PresomiticMesoderm/Somite0-eigen.mha
0.7

12. Apply the tensor voting filter to the output of the planarity filter - The source code for this filter is located at *MR-Source/Code/Examples/MembraneVotingField3D.cxx.* There are four command-line inputs - the planarity filter output, the eigen matrix image, the tensor voting output, and the voting neighborhood radius.

$ ./membraneVotingField3D DatasetS1/PresomiticMesoderm/Somite0-planarity.mha
DatasetS1/PresomiticMesoderm/Somite0-eigen.mha
DatasetS1/PresomiticMesoderm/Somite0-TV.mha 1.0

13. Use the watershed segmentation filter to obtain cell segmentations. Here, the input parameters consist of the preprocessed input image (for computing image gradients), tensor voting image, and the output segmentation image. We set a threshold value of 1.0 on the tensor voting data to differentiate background and foreground.

$ ./membraneSegmentation DatasetS1/PresomiticMesoderm/Somite0-preprocess.mha

DatasetS1/PresomiticMesoderm/Somite0-TV.mha
DatasetS1/PresomiticMesoderm/Somite0-segment.mha 1.0

2.5 Important parameter settings

In our code, there is only a single parameter (σ, Ω) to set for each filter, namely the neighborhood radius. This parameter is set in physical units and represents the radius within which to compute the planarity function and perform the voting operation. In the above example, we chose to use a neighborhood radius of 0.3 μm for preprocessing, $\sigma = 0.7\mu m$ for planarity filtering, and $\Omega = 1.0\mu m$ for tensor voting respectively.

3 Validation: Generating Synthetic Membrane Data

Synthetic $3D$ images are generated using the k-means algorithm [5] for generating centroidal Voronoi tessellations of the image region. These images provided as Dataset S2. The sequence of steps for generating similar such datasets are as follows:
(i) Select the number of cells ($k = 1000$) and randomly initialize their center positions within a volume (Ω) sampled by a high resolution grid of dimensions $500 \times 500 \times 500$ with pixel spacing of 0.2μm in x,y and z.
(ii) Run the k-means algorithm until convergence when cell centers stop changing their positions. At convergence, cells uniformly occupy the image region and roughly have equal volumes.
(iii) Compute a point cloud density function (M) representative of membrane markers at the Voronoi boundaries of cells as:

$$M(\mathbf{p}) = \delta(|d_i(\mathbf{p}) - d_j(\mathbf{p})|) \tag{3}$$

where δ is the Dirac-delta function impulse function and d_i,d_j are the distances to the nearest pair of cell centers.

(iv) The point spread function of the microscope is sumulated by convolving with a $3D$ Gaussian kernel ($G_{\sigma_x,\sigma_y,\sigma_z}$) as point spread function ($\sigma_x = \sigma_y = \sigma_z/2 = 0.2\mu m$), an image ($I$) is reconstructed on a new grid of spacing $0.2\mu m$, $0.2\mu m$ and $1.0\mu m$ in x,y and z respectively.
(v)The image is then corrupted with zero mean additive Gaussian (N_σ) and Poisson (P_s) noise distributions. Note that the Poisson noise distribution is dependent on the underlying image intensity as its mean. The s refers to the scaling of image intensities prior to generating noise which is the same as inverse of the gain applied during acquisition. The resulting image can be obtained using the following expression:

$$I(\mathbf{p}) = \int_\Omega G_{\mathbf{p},\Sigma} * M dx + N_\sigma(\mathbf{p}) + P_s(\mathbf{p}) \tag{4}$$

where $\Sigma = diag(\sigma_x, \sigma_y, \sigma_z)$.

In the folder Scripts, we have supplied a bash script file SyntheticMembraneGenerationBashScript.sh. Upon execution, this script creates two directories *validation/manual* and *validation/raw*. It then automatically runs a synthetic membrane image generator to generate a sequence of ten $3D$ images with varying (σ, λ) values. Three images spanning the range from (0.01, 1.00) to (0.1, 0.1) are shown in Figure S3. The folder *validation/manual* will contain the ground-truth segmentation images and *validation/raw* (Figure S3(a-c), for example) will contain simulated microscopy images. The idea is to apply our segmentation pipeline (steps 9-12) and compare with the segmented data to create Table 1 automatically.

4 Materials and Methods

4.1 Ethics Statement

Zebrafish were raised in an American Association of Laboratory Animal Care accredited fish facility following the guidelines outlined in the Guide for the Care and Use of Laboratory Animals, The Zebrafish Book, and those of the Harvard Medical Area Standing Committee on Animals.

4.2 Image acquisition

Fluorescent labeling of zebrafish embryos was conducted by injecting 1-cell stage embryos with 2.3nl of 40ng/μl each H2B-EGFP and mem-mCherry mRNA and were screened for health and brightness before imaging. For the somite data, embryos transgenic for nuclear localized tomato and membrane localized citrine (*Tg(actb2:Hsa.H2B-tdTomato)hm25; Tg(actb2:mem-citrine)hm26*) were used. Embryos were staged and mounted as described in [6,7]. Live imaging was performed using a Zeiss 710 confocal/2-photon microscope with a home-made heating chamber and C-Apochromat 40X 1.2 NA objective. Chameleon laser line 1020nm was used for 2-photon imaging and 488nm, 514nm, 561nm and 594nm for confocal.

References

1. Guy G, Medioni G (1996) Inferring global perceptual contours from local features. International Journal of Computer Vision 20: 113-133.

2. Medioni G, Kang SB (2004) Emerging Topics in Computer Vision. Upper Saddle River, NJ, USA: Prentice Hall PTR.

3. Loss L, Bebis G, Nicolescu M, Skurikhin A (2009) An iterative multi-scale tensor voting scheme for perceptual grouping of natural shapes in cluttered backgrounds. Journal of Computer Vision and Image Understanding (CVIU) 113: 126-149.

4. Parvin B, Yang Q, Han J, Chang H, Rydberg B, et al. (2007) Iterative voting for inference of structural saliency and characteriztion of subcellular events. IEEE Transactions on Image Processing 16: 615-623.

5. Du Q, Faber V, Gunzburger M (1999) Centroidal voronoi tessellations: applications and algorithms. SIAM Review 41: 637-676.

6. Megason SG, Fraser SE (2007) Imaging in systems biology. Cell 130: 784–795.

7. Megason SG (2009) In toto imaging of embryogenesis with confocal time-lapse microscopy. Methods in Molecular Biology 546: 317–332.
